# A Data-Driven Approach to Reverse Engineering Customer Engagement Models: Towards Functional Constructs

**DOI:** 10.1371/journal.pone.0102768

**Published:** 2014-07-18

**Authors:** Natalie Jane de Vries, Jamie Carlson, Pablo Moscato

**Affiliations:** 1 The Priority Research Centre for Bioinformatics, Biomarker Discovery and Information-Based Medicine, The University of Newcastle, Newcastle, New South Wales, Australia; 2 School of Electrical Engineering and Computer Science, Faculty of Engineering and Built Environment, The University of Newcastle, Newcastle, New South Wales, Australia; 3 Newcastle Business School, Faculty of Business and Law, The University of Newcastle, Newcastle, New South Wales, Australia; University of California, Irvine, United States of America

## Abstract

Online consumer behavior in general and online customer engagement with brands in particular, has become a major focus of research activity fuelled by the exponential increase of interactive functions of the internet and social media platforms and applications. Current research in this area is mostly hypothesis-driven and much debate about the concept of Customer Engagement and its related constructs remains existent in the literature. In this paper, we aim to propose a novel methodology for reverse engineering a consumer behavior model for online customer engagement, based on a computational and data-driven perspective. This methodology could be generalized and prove useful for future research in the fields of consumer behaviors using questionnaire data or studies investigating other types of human behaviors. The method we propose contains five main stages; symbolic regression analysis, graph building, community detection, evaluation of results and finally, investigation of directed cycles and common feedback loops. The ‘communities’ of questionnaire items that emerge from our community detection method form possible ‘functional constructs’ inferred from data rather than assumed from literature and theory. Our results show consistent partitioning of questionnaire items into such ‘functional constructs’ suggesting the method proposed here could be adopted as a new data-driven way of human behavior modeling.

## Introduction and Theoretical Background to the Study

Online consumer behavior has seen an increasing amount of interest by scholars and marketers alike. Consumers' changing behaviors online and new ways of communicating with one another and brands have provided many new implications for marketers [1]. Non-transactional consumer behaviors such as online or offline customer engagement have been of particular interest to scholarly and professional consumer researchers [2]. Empirical research in the area is emerging [3], [4], however, research on finding a robust and generalizable framework of online customer engagement and its related constructs is currently still limited. Furthermore, current research in the area takes the methodological approach of hypothesis testing researcher's queries with a priori parameters in place. However, hidden structures, correlations and relationships between variables may exist within any set of data [5]. Thus, the present study aims to learn from consumer research data and provide a ‘reverse-engineered’ model of online Customer Engagement (CE) and its related constructs. In doing so, we propose a novel methodology based on the premises of symbolic regression analysis, graph theory and finally, community detection within a graph. In this section a background on the relevant theory and literature is provided, followed by the methodology of our study, a presentation of the results, and finally a detailed discussion of the proposed method and findings and directions for future research.

Although interest on the topic has grown, confusion amongst marketers and scholars exists regarding the construct of CE, particularly in the online environment. Conceptual research in the field argues that, through CE, customers are able to collaboratively create a better experience for themselves whilst engaging with the brand, create more value and interact with the brand in order to identify and understand problems and develop solutions for themselves [6] [7] [8]. A common online social media platform to facilitate CE, Facebook, defines engagement as ‘*The unique number of people who liked, commented, shared or clicked on your posts*’[9] which has a greater focus on the actual behaviors customers display towards brands on the social media platform. Recent empirical studies have used this definition for their analysis of online CE with brands (e.g. [4]) and have therefore focused on their very Facebook-specific behaviors in terms of customers interacting with the brand and one another. However, other recent scholarly research defines CE differently and more broadly. The definition of CE brought forward by Van Doorn et al. [2] has been cited by multiple other scholars in the field such as Jahn and Kunz [3]. It defines CE as the behaviors that go beyond transactions including a customer's behavioral manifestations that have a brand or firm focus, beyond purchase, resulting from several motivational drivers has. Furthermore, scholars argue that CE refers to a psychological state reflecting customers' interactive, co-creative experiences with a focal object (such as a brand or an object that personifies the brand like a brand's website) which highlights the active role of the consumer [10], [11].

The above definition and description of CE takes the view that ‘engagement’ is psychological assessment with a behavioral orientation and therefore focuses on the customer's perceived integration with a brand object such as a firm's online brand community within branded social media pages rather than the specific behaviors displayed through the online platform. Furthermore, the construct Jahn and Kunz [3] utilized to measure online customer engagement included items such as the level of the customer's perceived ‘integration’ within the online brand community, the level of ‘engagement’ felt by the customer within the online brand community, the level of that customer's ‘activity’ within the online brand community, the level of ‘participation’ within the online brand community and finally, the level of ‘interaction’ within the online brand community on the social media platform. In contrast to this, the empirical research taking Facebook's definition of actual ‘engagement behaviors’ looks at the actual number of likes, the frequency of comments and the actual number of times the content uploaded by the page is shared by Facebook users [4]. These differing ‘measurement items’ further emphasize the different views of CE as either being a psychological assessment by the customer, or as actual behaviors displayed in terms of liking, sharing and commenting on the brand's online content.

With such discrepancies in the definition of the CE construct, the inclusion or exclusion of specific measurement items to measure a customer's perceived level of online CE with a brand is open for discussion and investigation. Furthermore, ambiguity around the motivational drivers and consumer behavior outcomes of online CE exists within the literature as several relating constructs have been proposed. Scholars argue that the relationship between the customer and the brand is central for online CE [12] and therefore it could be argued that the previous relationship between a customer and a brand impacts that customer's level of engagement with that brand online. As part of the existing customer-brand relationship, brand involvement [13] and self-brand congruency [14] have been related to online consumer behaviors towards a brand. Furthermore, constructs based on the theory of Uses and Gratifications (U&G) have been found to be part of the process and motivational drivers of CE [3]. Also, particularly in the online environment, the concept of ‘Flow’ has been introduced as having an impact on consumers' attitudes and behaviors towards a brand (e.g. [15] [16]) and could therefore also be relational to online CE. In addition to this, the notion of customer Co-Creation of Value (CCV) leading to higher levels of CE has been introduced by Sawhney et al. [7] and aligns with the view of those scholars who see online CE as a way for the customer to create more value and interact with the brand and solutions for themselves (e.g. [6] [8]). This means that there are many possible constructs related to or driving online CE proposed by the literature and a defined model or relational process of online CE is currently still being evolved.

A common method for social scientists, including marketers, to conduct research is through hypothesis and significance testing [17]. In this manner, hypotheses are proposed based on theory or prior knowledge and commonly a study is designed using questionnaires, interviews or secondary data collection followed by either modeling or analysing this data and finally providing statistical testing of the proposed model [18]. However, in practice, marketing and brand managers are of the opinion that decision-making needs to be data-driven in order to make more valuable decisions [19]. Other scientific disciplines, such as computer science, sociology and physics for instance, systematically use a data-driven methodology in order to find unknown trends, patterns or other answers to unknown or un-defined problems. For example, a study on the inference of transcriptional gene regulatory networks [20] found that data-driven community-based methods provide a powerful and robust tool. Furthermore, “reverse-engineering” of causality models or, inference investigation, has also been conducted in studies regarding systems-biology networks [21], gene regulatory networks [22], high-dimensional plant gene expression data [23] and in other multidisciplinary studies like this one such as [24], which examines social and biological studies. Frequently, this type of research yields interesting results with often unexpected findings as the researcher *learns* from the data [25] rather than only testing the statistical validity of a certain model given the data.

Hence, as stated, the present study intends to learn from consumer behavior data and provide a ‘reverse-engineered’ model of online CE and its related constructs. Such a novel methodology could provide researchers in the fields of marketing and other consumer and human behaviour studies a new method for finding models that are inferred from data, rather than hypothesised based on existing literature. ‘Reverse-engineering’ a model from data is a practice found in science in order to gain a better understanding and find robust results [26], [27]. Analogously, this study analyses survey data obtained in a questionnaire that contained a varied list of constructs including and relating to CE. Although the items included in this questionnaire are informed from existing theory, the method proposed by this study aims to find results that appear naturally from this data rather than set parameters of the model a priori. In taking this approach, this study provides the opportunity to analyse online consumer behavior data in a ‘different direction’. Rather than testing hypotheses, this study infers correlations and patterns from the data.

The manner in which this study ‘learns from data’ is through the use of mathematical optimization techniques. In a similar fashion to other reverse engineering methodologies, we aim to find a model that achieves a high level of prediction of each of the variables having as input all the other variables via an approach based on *symbolic regression* [28]. We use a powerful software named *Eureqa* [29] that is based in Evolutionary Computation techniques to search for the best linear model. We also investigate the results that could be inferred by working with a data set with half the sample size. We investigate this possible scenario through randomly separating the whole data sets into two age and cardinality matched subsets; Set A and Set B and following the same process for each. We then use mathematical methods from graph theory and network analysis to identify groups of variables that co-segregate in “communities” and link them with the notional definition of constructs.

Part of our motivation that supports our strategy of mathematically modeling each individual item lies in the possible ‘hidden’ constructs within the data which may be made up of items from multiple ‘theoretically-formed’ constructs. For example, the construct of ‘customer engagement’ could include items from other constructs which more accurately measure that customers' level of engagement with the brand such as ‘brand involvement’ or the level of actual interaction between the customer and the brand page. We suggest in this study that those ‘hidden constructs’ found through mathematical modeling, may form ‘*functional constructs*’ through a combination of several items. Furthermore, other related constructs seen as two separate constructs, such as ‘brand involvement’ and ‘self-brand congruency’ may actually measure consumer behavior more accurately when combined into one construct. Finding out whether the items from each construct are more correlated with items from other constructs could provide interesting results for practice and theory in measuring online consumer behaviors. Therefore, one of the objectives of this study is to discover new (functional) and existing constructs within the data through quantitative analysis. As stated, whilst doing so, a novel methodology will be proposed and evaluated which will provide research with future opportunities of data analysis through reverse-engineering a model in consumer behaviour studies.

The structure of this article is as follows. In the Methods section the proposed methodology is presented as a five-stage process, explaining the details of each of its stages including an ethics statement and an outline of the data collection process. In the results section we present the findings of each stage of the study including the statistical evaluation of the outcomes. Finally, supported by our results we provide unifying discussions, including the limitations of the study as uncovered by our findings and we provide our final conclusions including possible future work in this evolving field.

## Method

**Figure 1:**
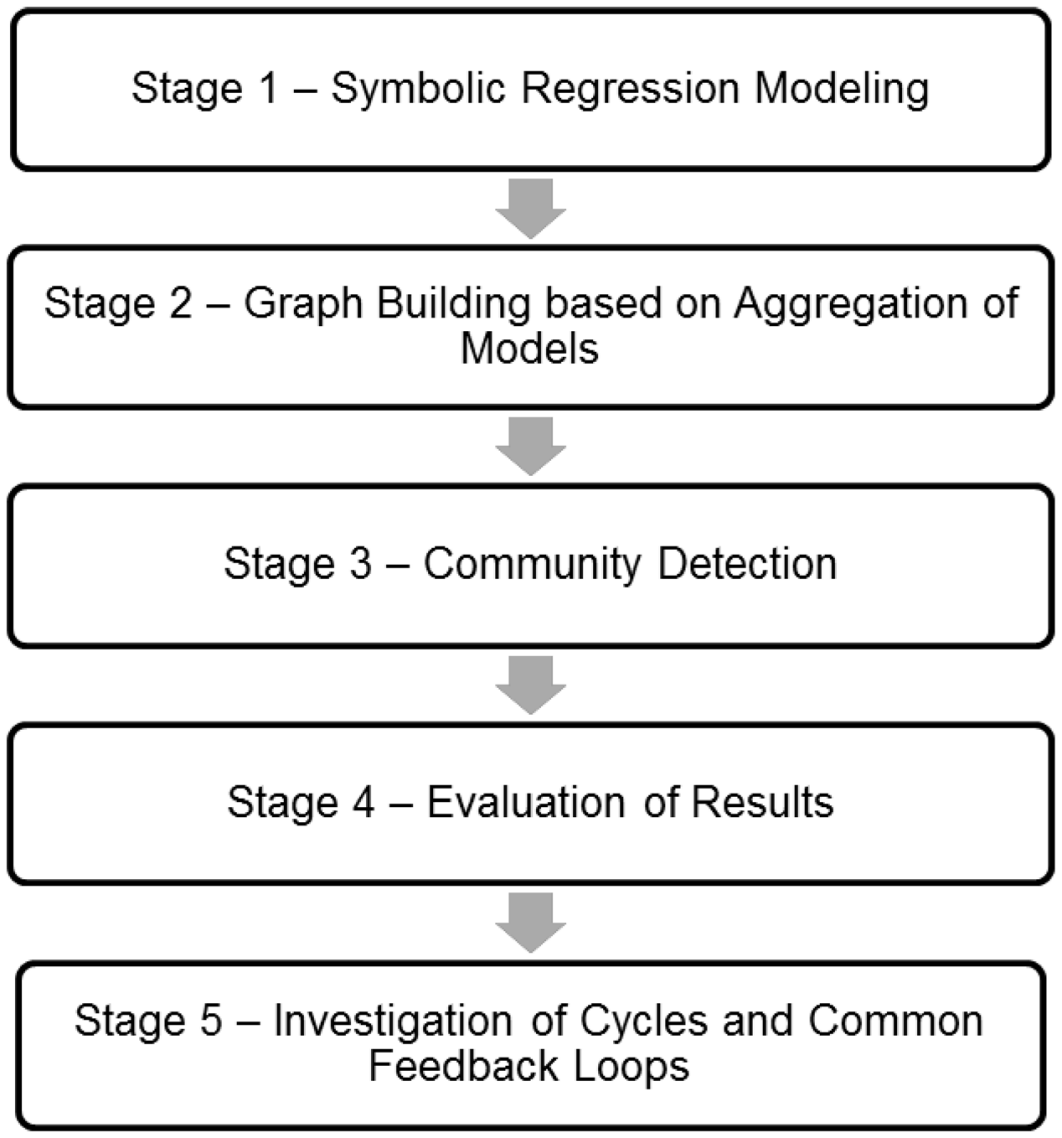
Stages of Research Methodology.

In this article we propose a data-driven approach to finding online consumer behavior constructs based on the correlation of individual items through mathematically modeling graph theory and network analysis. In doing so we aim to put forward a new method for scholars to analyse their data which can provide an alternative to research based on hypothesis testing. The process of this novel methodology is carefully outlined in this section and presented in Figure 1. The data used is primary data obtained through data collection with pen-and-paper questionnaires. We provide an ethics statement, a detailed description of the data collection process and outline the preliminary data analysis method used. Finally, the proposed method including each stage is carefully outlined and described.

### Ethics Statement

This study was approved by the University of Newcastle ethics committee (Ethics Approval Number: H-2013-0226) (this governing body is equipped with an ethical review board). The questionnaire tool was anonymous and no identifying information was asked of participants. Prior to participation, participants were given a Participation Information Statement to read with all necessary information. Participation was entirely voluntary with no incentive given and participants provided their implied consent by completing the survey and were able to discontinue their participation at any time whilst completing the survey.

### Survey Sites and Questionnaire Development

The data collection method for this study was conducted through a questionnaire survey at selected sites within the University of Newcastle campuses. Respondents for the survey were recruited via both an intercept on the Callaghan University Campus in non-learning areas (e.g. cafeterias) and in selected business and marketing courses on the Callaghan and Ourimbah campuses within the time frame of three weeks. Considering the usage profile of the general Facebook user being a young adult between the age of 18–24 [30], it can be contended that a sample of Undergraduate University students is appropriate due to the similar demographic profile. The questionnaire tool was constructed using existing measurements adopted from relevant academic literature in the areas of consumer behavior and services marketing and is provided in File S1. The paper-based surveys were administered to Facebook users only who had previously used a Facebook brand page. In the questionnaire, each item was measured on a seven-point Likert scale anchored with (1)‘*strongly disagree*’ to (7) ‘*strongly agree*’ which is consistent with previous online consumer behavior studies on social networking sites e.g. Jahn and Kunz [3] and other internet web-based studies e.g. Carlson and O'Cass [13]. A file containing the completed answers to the questionnaires are provided in File S2. For the remainder of this article we will refer to items by their code given which have been outlined in Table 1 as well as the source of where the construct was adopted from and the number of items included in each theoretically-driven construct. The total number of items included in the questionnaire equals 69.

**Table 1:** Theoretical Constructs Description and Source.

Construct	Source	Code	No. of Items
Usage Intensity	Jahn & Kunz (2012) [3]	UI	3
Brand Involvement	Adapted from Carlson & O'Cass (2011) [13]	INV	6
Self-Brand-Congruency	Adapted from Hohenstein et al. (2007) [14]	SBC	5
Functional Value	Jahn & Kunz (2012) [3]	FUV	4
Hedonic Value	Jahn & Kunz (2012) [3]	HED	4
Social Interaction Value	Jahn & Kunz (2012) [3]	SOC	4
Co-Creation Value	Adapted from O'Cass & Ngo (2011) [55]	CCV	6
Customer Engagement	Jahn & Kunz (2012) [3]	CE	5
Customer Loyalty	Jahn & Kunz (2012) [3]	LO	6
SNS-Specific Loyalty Behaviours	Adapted from O'Cass & Carlson (2012) [56]	ON	3

### Preliminary Data Analysis

After the data collection of 452 surveys, 371 useable surveys remained after screening and data cleaning. Preliminary data analysis has been conducted using the Statistical Software SPSS v21. Of this sample, 41.8% were males and 58.2% were females. The age ranged from 17 to 49 with an average age of 21.40. Data was screened using SPSS to establish means, skewness and standard deviations in order to develop a general understanding of the data set and to ensure the data suited further analysis (i.e. checking if the data was non-normally distributed). Skewness is defined as a characteristic of a distribution that assesses its symmetry around the mean [18], [31]. Negative skewness values indicate a negative skew versus a positive skew for positive skewness values. The Skewness values in the whole data set fall within the range of +2 to −2 which means that we are able to assume the data is normally distributed [32]. For the purposes of this study, the data was randomly split into two subsets; Set A and Set B to act as a validation set of the results. These two subsets have also been analysed in terms of descriptive statistics. Set A comprised of a total number of 185 samples of which 45.9% were males and 54.1% were females. The age within Set A ranged from 18 to 49 with an average of 21.54, similar to the average age of 21.4 of the whole data set. Set B comprised of a total number of 186 samples of which 37.6% were males and 62.4% were females. The age range within Set B is age 17 to 46 with an average age of 21.26, which is also similar to the average of the whole data set at 21.4. Furthermore, the skewness values for all variables in both Set A and Set B fell within the range of −2 to +2 which mean normality of the data is satisfied.

### Design of the Study

The design of the study has been motivated by scientific research which is data-driven, particularly in the area of reverse engineering biological networks from experimental data (see for instance [27]). Towards this end, we have used a suite of computational methods in public domain software. First, each data set is analysed through symbolic regression modeling each of the 69 items. This gives mathematical models based on correlation between two items. Based on the aggregation of the results of this mathematical analysis, a graph is formulated and visualized. Within this graph, a method of community detection; modularity optimization, is computed to find ‘communities’ of items which may possibly form ‘functional constructs’. In this study, we propose the concept of ‘functional constructs’ meaning those communities of items that will emerge as a result of the community detection through modularity optimization. Finally, the results of the study are assessed using multiple assessment criteria suitable in order to evaluate the findings of this study. This process is outlined in Figure 1 and described in further detail in the remainder of this section.

### Stage 1 Symbolic Regression Modeling

Given that the aim of this study is to find results that are data-driven, this study employs the method of symbolic regression. Unlike “normal regression” methods, where model hypotheses are generated and fit to available data, symbolic regression involves the discovery of the structure as well as the coefficients within that structure [5]. As Smits and Kotanchek [5] explain, a significant problem with implementing symbolic regression is the difficulty in making the trade-off between expression accuracy and complexity. First introduced by Koza, symbolic regression is generally done via genetic programming and is defined as ‘finding a mathematical expression, in symbolic form, that provides a good, best or even perfect fit between a given finite sampling of values of the independent variables and the associated values of the dependent variable(s)’ [33]. In simpler terms, symbolic regression involves finding a model that fits a given set of data. Importantly, an advantage of genetic programming methods for symbolic regression is that unlike with numerical regression methods, the user does not have to specify the form of the regression model in advance [34].

Making the method proposed in this study easily adoptable for future research, open access tools and software packages are used for analysis. *Eureqa* [29] is a mathematical symbolic regression tool with a clear user interface which is free for academic use. The output of *Eureqa* is a Pareto optimal curve that trades model fitting for its complexity. We have selected the best fitting linear regression model in this front. This said, *Eureqa* is utilized in this study by finding linear regression functions for each item that balance the trade-off between model fitting and complexity. Each individual item was placed as the target variable (i.e. to be the dependent variable) and regression with a Pearson correlation coefficient as error metric was run a total of 69 times in order to find a model of each item as a function of the others. Although symbolic regression is a method in which a limited number of a priori assumptions are made, appropriate functional building blocks need to be selected by the researcher [5]. *Eureqa* allows the user to select such building blocks for the possible solutions. For this study, the following building blocks were selected: *constant, integer constant, introduction of an input variable, addition, subtraction* and *multiplication*. In addition, we chose the option in which all data points were treated equally. After this user selection, *Eureqa* runs its evolutionary search procedure to find the solution that fits the data best with the lowest possible level of complexity as is consistent with symbolic regression methods [28]. For the purpose of the present study, the process was stopped after no new best linear regression equation was found for a period longer than 60 seconds and the best-fitting linear regression solution was selected. As *Eureqa* evaluates millions of possible combinations during that time, we have found that in practice this has been an acceptable limit for the problem at hand. The relationships of the variables that were found to be correlated with the target variable are represented by the connections in the visualized graph as outlined in Stage 2.

### Stage 2 – Graph Building based on Aggregation of Models

After the initial process of symbolic regression in the *Eureqa* software, the putative functional relationships found between questionnaire items were scribed into an interaction model that formed into a graph. That is, all relationships between the 69 individual items were aggregated and drawn to create a graph. Completing this graph by iteratively running *Eureqa* using all the items as targets for model identification will finally form a network of connected nodes and arcs (a directed graph). Graphs are defined simply as discrete structures that consist of vertices (or nodes) and edges that connect these vertices [35]. Edges may be directed or undirected, weighted or unweighted and may carry a ‘capacity’ [36]. Generally, when the relationship implies a direction, it is referred to as an arc. In this study, each node within the graph represents a questionnaire item which means that the graph contains 69 nodes and an ‘arc’ represents a relationship found between those two items through the symbolic regression iterations *Eureqa* (if an arc goes from *a* to *b* this means that question *a* has been found as input variable in one of the best linear regression functions that “explain” *b*. However, in the next stage of the proposed method, community detection of items through the optimization of modularity, we consider the network as a simple graph (unweighted and undirected) so as to keep the reproducibility of this study possible using free software tools.

### Stage 3 – Community Detection in graphs via Modularity Optimization

At a very basic level, a network can be defined as an undirected graph: a set of nodes or vertices connected in pairs by lines or arcs, but may be much more complex or contain many variations [37]. Networks are found to divide naturally into communities described as sets of highly interconnected nodes [38]. An effective approach in detecting and characterizing a community structure is the optimization of the quality function of ‘modularity’ over the possible partitions of a network [39]. As Newman [39] describes, the nodes in many networks fall naturally into groups (or communities), sets of nodes among which there are many arcs, with only a smaller number of arcs between nodes of different communities. In other words, ‘in the standard formulation of modularity, a sub-graph is a community if the number of arcs within the sub-graph exceeds the expected number of internal arcs that the same sub-graph would have in the null model. This expected number is an average over all possible realizations of the null model.’ [40].

Modularity is a global criterion to define a community; a quality function and the key ingredient of the most popular method of graph clustering [40]. As Fortunato [40] states, by assumption, high values of modularity indicate good partitions of modularity classes (or communities) within network graphs. The optimization of modularity is seen as being part of the most successful solutions to the community detection problem [41]. In the present study, we use the modularity optimization algorithm brought forward by Blondel et al. [38] in order to partition a network into communities or ‘sub-groups’ of nodes. This algorithm is available for use in a free software visualization tool: *Gephi* [42]. A high modularity score for a data set indicates a “sophisticated” internal structure and these scores will be reported with the results of our study. The optimization of modularity as a community detection method has several advantages. It has been found that in terms of computation time, modularity is favorable and moreover, the quality of the findings from modularity optimization is found to be of a high standard [38].

Based on Blondel et al.'s [38] algorithm for modularity optimization, the network graph is divided into groups (or communities) and results are displayed in the following section. Next, the communities based on modularity were selected as a partition parameter and represented in the graph through identifiable colours. These communities of individual items now represent sets of variables within the data which collectively seem to be influencing other sets of variables. The communities between Set A, Set B and the whole data set are compared for consistency in number of classes found, number of items within each community and the number of similar items between the communities in the three data sets. Also, a comparison is made in regard to the number of, and the target and source of, the arcs within each graph. The reason for doing so is to provide a more thorough comparison of each data Set and its properties.

### Stage 4 – Evaluation of Results

Several measures have been used in this study in order to assess the quality of our results. Firstly, the analysis of the communities found in each set was done by creating contingency tables (displayed in the following section) comparing the modularity classes between Set A and Set B, between Set A and the Whole Data Set and between Set B and the Whole Data Set. The number of similar items in each group is counted in order to compare both sets with each other and the whole data set. As stated, the number of arcs in each set is also compared and the results of this analysis are displayed in tables in the following section. In order to assess these contingency tables statistically, the values for *Cramer's V* will be computed. *Cramer's V* is a measure of the strength of association among the levels of the row and column variables [43]. Given the rows in our contingency table represent the communities from one set and the columns the communities from another set, the *Cramer's V* will provide an indication of the strength of the association between communities in each set.

Finally, further evaluation analysis of the community partitioning is done by using two more measures; the *Adjusted Rand Index* and *Fleiss' Kappa*. The Rand Index, or more specifically, the *Adjusted Rand Index* (ARI) proposed by Hubert and Arabie [44] is frequently used in cluster validation that involves non-overlapping groups, as in this case, since it is a measure of agreement between two partitions: one given by the clustering process (in this case: modularity partitioning process) and the other defined by external criteria [45]. In this study the way the items were partitioned into communities, or ‘modularity classes’ in each of the data sets is evaluated through the ARI.

Furthermore, the *Fleiss' Kappa*, is a measure of ‘interrater agreement’ which measures the degree of agreement when multiple ‘raters’ are present (in this case multiple data sets for the same experiment). As Fleiss, Levin and Paik [46] explain, when the level of agreement is high, then there is a possibility that the different ratings do reflect the dimension they are supposed to reflect. In this case, we aim to evaluate the level of ‘agreement’ between the data sets and the way in which the data has been partitioned in each. On the other side, if the level of agreement is poor, the opposite is true and in the case of this study would mean a bad reflection on the separation of modularity classes in the two data sets. In this study, we provide a pairwise agreement between Set A and Set B, Set A and the Whole Set, Set B and the Whole Set and a Kappa measure for all three together. Results for this measure are provided after experiment results.

### Stage 5 – Investigation of Cycles and Common Feedback Loops

In a given graph with connected nodes and edges (or arcs), it can be expected that this graph is not acyclic. That is, it is likely that cycles, or ‘feedback loops’ are present in the graph. Following a data-driven approach, motivated by research in varying sciences, it is natural to investigate the cycle structure in the graph we have generated in Stage 2 and 3 since it may indicate any possible feedback loops that could have been uncovered by our study. Finding such feedback loops or ‘cycles’ in networks is already a common practice in many sciences. Recently, marketing researchers have begun to explore ‘feedback effects’ in models of consumer’s reciprocal evaluations of parent brand equity [47]. The baseline approach is to directly compare the common cycles present in the three data sets. In doing so, it would be useful to first explore the common arcs between the graphs as it is the arcs that are going to show possible feedback loops.

However, finding the common cycles in graphs through examining the common arcs is a computationally hard problem considering the graph resulting from Set A contained 264 arcs, Set B contained 239 arcs and the graph for the Whole Data Set contained 250 arcs. Due to this being a computationally hard problem, we present a relatively simpler and heuristic approach to cope with this problem. The nodes that are part of the same theoretically-driven construct and part of the same community based on modularity and connected via an arc, are ‘merged’ into one node. For example, in one case all five items relating to Customer Engagement (ENG1 – ENG5) have appeared together in one community and were all connected. In this case these nodes will be merged in one single node showing the relationships of customer engagement (ENG) as a whole to other nodes. This also means that duplicate arcs are merged to indicate the relationship between variables as a whole, rather than between each individual variable. This process significantly reduces the size of the graph without losing information. This process is undertaken for each of the graphs, for Set A, Set B and the Whole Set. After this, we aim to investigate the cycles that are common between the three sets. Results are displayed in the respective Stage 5 in the results section and further elaborated in the discussion section.

## Results

In this section, we present the results obtained with the proposed method based on symbolic regression, graph theory and community detection through modularity optimization. Also, as stated in our proposed methodology, an assessment of the results is provided. This section is split up to show the results of each stage of the study.

### Stage 1 – Symbolic Regression Modeling Results

**Figure 2:**
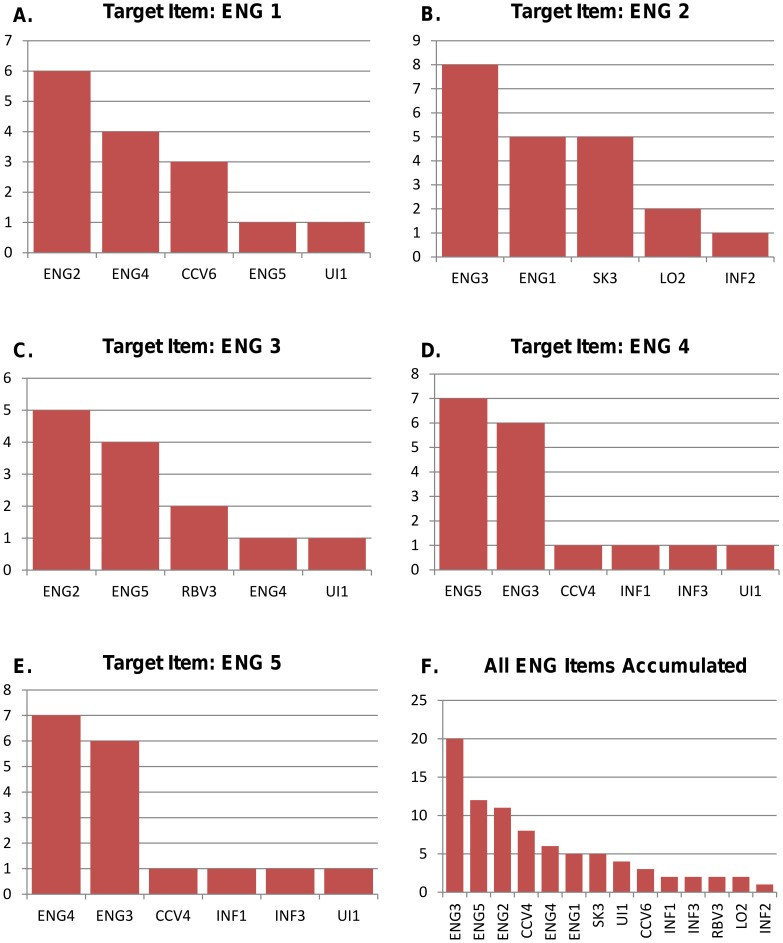
The Figure shows the items ‘used’ by *Eureqa* through symbolic regression setting each of the five ENG items as dependent variables (obtained using the whole data set). Each red bar shows the number of times that particular item appeared. Clearly, the high correlation of the ENG variables among themselves is found, however in the accumulated results we observe the conspicuous role of other items as shown in Figure f. N.B. The full results for each item (n = 69) and each construct can be made available as supplementary material upon request.

As outlined in the previous section, through symbolic regression we aim to find the best linear regression model that best fits the data for each individual item. As the questionnaire included a total 69 items, and the process was run iteratively for each of these items, not all regression models and their results are presented. In this study, the construct of particular interest was that of Customer Engagement (ENG) including the items from the proposed construct by Jahn and Kunz [3]. Figure 2 displays the results of each individual item relating to the Customer Engagement construct (ENG1 – ENG5). The bar charts summarize the number of models proposed by *Eureqa* each individual item appeared in. In other words, *Eureqa* keeps track of how many times it ‘uses’ a variable in its proposed set of solutions in the optimal Pareto front [29] [48]. Although the items included in these results may not all feature in the best-fitting linear model selected for the purpose of this study, these results give a clear indication of the interactions between items of varying constructs. Figure 2 shows the number of times a single variable was used by *Eureqa* in possible solutions for each of the ENG items in the Whole Data Set. For all customer engagement items, the most commonly used item (and therefore suggested by *Eureqa* to be the most correlated) was another customer engagement item. Overall, the most commonly used item for all models was that of ENG3. ENG3 measures a customer's level of *activity* within the brand page with the following statement: ‘*I am an active member of this Facebook brand page community*’. This could indicate that within the Customer Engagement construct as marketing literature has proposed it, this item could be considered, based on this data and methodology, as the most central or fundamental item. Furthermore, items CCV4, CCV6, SK3, UI1, INF1, INF2, INF3, RBV2 and LO2, belonging to the the CCV (Co-Creation Value), SK (Subjective Knowledge), LO (Loyalty), INF (Informational Value), UI (Usage Intensity) and RBV (Relationship-Building Value) constructs are also included in possible *Eureqa* solutions for ENG items indicating some sort of direct influence to these items. The full statements of these items can be found in the survey questionnaire.

### Stage 2 & 3 - Graph Building Based on Aggregation of Models - Community Detection

**Figure 3:**
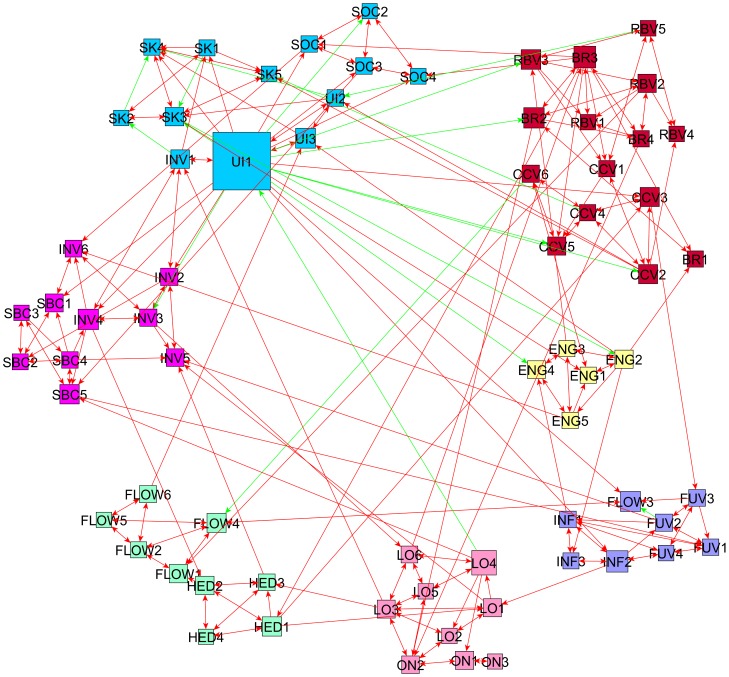
Data Set A – Network found as a result of the application of the model finding optimization software on each variable as a target. This is a directed graph in which an arc connects a variable that appears as an input in a model for a target variable. There is a clear association of variables that belong to the same construct as shown by their labels but other “functional constructs” combining items from several different theory-driven consructs emerge. The size of a node is proportional to its centrality in the network measured by ‘*node betweenness centrality*’ A green line represents a negative relationship found and a red line a positive relationship between the two nodes (items).

As outlined in the previous section, all relationships found through symbolic regression were scribed into a directed graph which formed a network of connected nodes and arcs. The results are visualized and displayed in this section. Furthermore, the visualizations also include the results of the community detection method through modularity optimization. In the graph visualizations, each node represents one of 69 individual items and each edge (arc) represents a correlation found by *Eureqa* between those two items. Subsequently, this network was partitioned based on modularity for each Data Set. The modularity classes have a higher than expected number of arcs between them, and a lower set of arcs with other classes (or communities). Figure 3 shows the network graph for Data Set A. As opposed to the initial constructs adopted from marketing literature which included 15 constructs, seven modularity classes (communities) were found.

The Customer Engagement items (ENG1 – ENG5) were grouped as one community showing that in Data Set A (*n* = 185), the Engagement construct as proposed by the literature, is also supported mathematically from the nature of existing correlations in the responses in the data. The largest community found in Set A included all items relating to the online interaction and relationship to the brand which represents a possible ‘functional construct’ as proposed by our methodology. The items included in this community relate to the constructs of; Relationship-Building Value (RBV1 – RBV5), Co-Creation Value (CCV1 – CCV6) and Brand Interaction Value (BR1 – BR4).

The items from the constructs of Social Value (SOC1 – SOC4), Usage Intensity (UI1 – UI3) and Subjective Knowledge (SK1 – SK5) form the second largest community and represent another possible ‘functional construct’ as inferred from the data. All items pertaining to the customers' previous relationship with the brand, that is; Brand Involvement (INV1 – INV6) and Self-Brand Congruency (SBC1 – SBC5) were also grouped into one community which formed the third largest group of nodes. Furthermore, both online and offline loyalty items (LO1 – LO6 and ON1 – ON3) were recognised to be similar and grouped into one community with in total nine nodes.

The majority of the items of the ‘Flow’ construct (FLOW1, FLOW2, FLOW4, FLOW5 and FLOW6) and all four Hedonic Value (HED1 – HED4) items formed one community, also in a group of nine nodes. Finally, similar constructs; Functional Value (FUV1 – FUV4) and Informational Value (INF1 – INF3) were grouped as one construct through modularity optimization with one ‘Flow’ (FLOW3) item added to this community.

**Figure 4:**
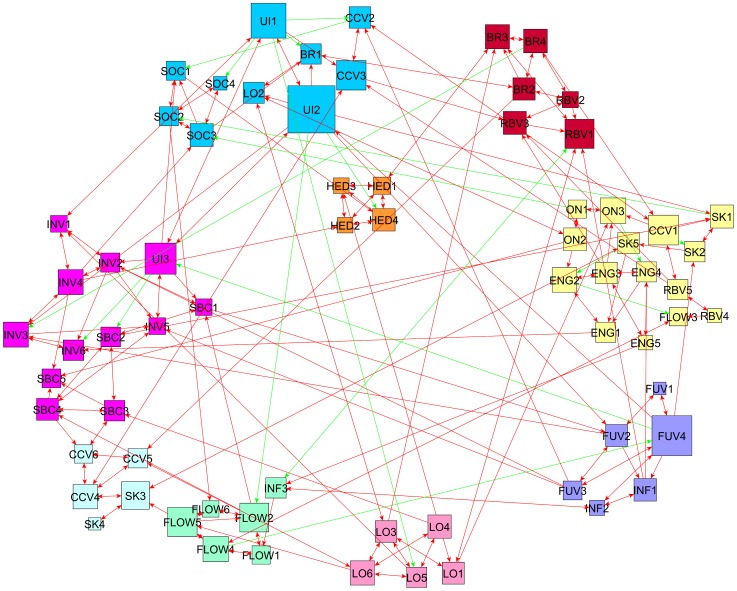
Data Set B – Directed network linking the variables with modularity classes computed with the same methodology as Figure 3. The community that contains variables of the constructs of Self-brand congruency and Brand Involvement (as well as one of the Usage Intensity) is again an example of a functional construct as found by the modularity optimization algorithm. The size of a node is proportional to its centrality in the network measured by ‘*node betweenness centrality*’ A green line represents a negative relationship found and a red line a positive relationship between the two nodes (items).

The same process was repeated for Data Set B (*n = 186*) which resulted in nine communities with a different organization between the groups as represented in Figure 4. This time, the Customer Engagement items are again together and now are paired with several other items which are: all three Online Loyalty items (ON1 – ON3), some Subjective Knowledge items (SK1, SK2, and SK5), two Relationship-Building Value items (RBV4 and RBV5), one Flow item (FLOW3) and one Co-Creation Value item (CCV1) which may suggest a large functional construct made up of many different items. This is also the largest community of items comprising of 15 nodes in total.

The second largest community comprises of 12 nodes and includes the variables relating to the customer's previous relationship with the brand: Brand Involvement and Self-Brand Congruency. All Brand Involvement items were included (INV1 – INV5), most of the Self-Brand Congruency items (SBC1 – SBC5) and one Usage Intensity item (UI3) was also included in this community. This community is similar to the one found in Set A where the majority of the variables relating to a customer's previous relationship with the brand were grouped together to form a large construct as informed from the data.

The third largest community with 10 nodes, included the other two Usage Intensity items (UI1 and UI2) as well as all four Social Value items (SOC1 – SOC4), two Co-Creation Value items (CCV2 and CCV3), one Loyalty item (LO2) and one Brand Interaction item (BR1). The remaining communities are of a smaller size with no class larger than 6 nodes. Similar to Data Set A, Functional Value (FUV1 – FUV4) items are grouped together with Informational Value items (INF1 and INF2), and most loyalty items are also grouped together without the online loyalty values (LO1, LO3, LO4, LO5 and LO6).

The items relating to Flow (FLOW1 – FLOW5) are all in one community together with one Informational Value item (INF3). Three of the Brand Interaction Value items (BR2, BR3 and BR4) are classed together with three of the Relationship-Building Value items (RBV1, RBV2 and RBV3) similar to the larger ‘brand-interaction’ class in Set A. The last two classes each contain four items; one with all Hedonic items (HED1 – HED4) which forms a very robust constructs as it is all of them are interconnected and finally, three of the Co-Creation Value items (CCV4, CCV5 and CCV6) together with two Subjective Knowledge items (SK3 and SK4) create a quasi-clique in a small group of nodes.

Again, the same process has been repeated for the Whole Data Set (*n = 371*) where seven communities of items were found, which corresponds with the number of communities found in Set A. The Customer Engagement items (ENG1 – ENG5) form as part of the largest group of items (15) in the Whole Data Set. Also included in this group are the Usage Intensity items (UI1 – UI3), the Subjective Knowledge items (SK1 – SK5) and two of the Brand Involvement items (INV3 and INV6) indicating again, as in Set B, that Customer Engagement may be measured as part of a larger ‘functional construct’ containing more items.

The second largest group of items comprised of 14 items and includes all Co-Creation Value items (CCV1 – CCV6), all Relationship-Building Value items (RBV1 – RBV5) and three Brand Interaction Value items (BR2 – BR4) which makes this group similar to the brand-related community of variables found in Set A. Next, there are two communities of the size of nine items. One comprises all the online and offline loyalty items (LO1 – LO6 and ON1 – ON3) and the other includes all Self-Brand Congruency items (SBC1 – SBC5) and the remaining Brand Involvement Items (INV, INV2, INV4 and INV5).

Furthermore, two communities with a size of eight items were found. One comprises of all Functional Value and Informational Value items (FUV1 – FUV4 and INF1 – INF3) as well as one of the Flow items (FLOW3), which makes this community of variables similar to the Functional and Informational value group found in Set A. The other group of eight items includes all Hedonic Value items (HED1 – HED4) and four Flow items (FLOW1, FLOW2, FLOW4 and FLOW5). Finally, the smallest community of items found in the Whole Data Set has 6 items, four of which are Social Value items (SOC1 – SOC2), one Brand Interaction Value item (BR1) and one Flow item (FLOW6) indicating yet another possible functional construct as inferred from the data.

### Stage 4 – Evaluation of Results

The comparison of the modularity classes (communities) and the number of common items within them is shown below. Table 2 depicts the contingency table of comparison between Set A and Set B communities. As stated, the number of communities of items in Set A equals to seven (*c*1A-*c7*A), whereas in Set B, nine communities (*c*1B-*c*9B) were found as shown in Table 2.

**Table 2:** Comparison of communities in Set A and Set B.

Community	*c*1B [6]	*c*2B [15]	*c*3B [10]	*c*4B [5]	*c*5B [6]	*c*6B [6]	*c*7B [12]	*c*8B [5]	*c*9B [4]
***c*** **1A [15]**	**6**	3	3	0	0	0	0	3	0
***c*** **2A [5]**	0	**5**	0	0	0	0	0	0	0
***c*** **3A [13]**	0	3	**6**	0	0	0	2	2	0
***c*** **4A [9]**	0	3	1	**5**	0	0	0	0	0
***c*** **5A [9]**	0	0	0	0	**5**	0	0	0	4
***c*** **6A [8]**	0	1	0	0	1	**6**	0	0	0
***c*** **7A [10]**	0	0	0	0	0	0	**10**	0	0

The values indicate the number of common items between two communities; highlighted in the main diagonal cells are the largest overlaps between the two sets. The numbers in parentheses indicate the number of items existent in each community or construct.

Furthermore, when the same process was undertaken for the whole data set, seven communities (*c*1W-*c7*W) were found. Set A had a total of 264 arcs, Set B had a total of 239, and the whole data set had exactly 250 arcs. In terms of modularity, Set A had the highest level of modularity (0.54) followed by the whole data set (0.495) and then Set B (0.486) as reported by Blondel's modularity optimization algorithm implemented in the *Gephi* software [49]. The Tables in this section report the counted number of the same items which were grouped to the same community. The diagonal emphasized cells indicate the matches between the most similar communities between Set A and Set B.

As shown in Table 2, similarities exist within the communities found in each set. Community one in each set comprised of 6 of the same items, community two of 5, community three of 6, community four of 5, community five of 5, community six of 6 and community seven of 10 common items. Furthermore, the eighth and ninth communities found in Set B had several similarities to the communities in Set A as shown in the Table. Upon further visual examination of the contingency tables, it becomes apparent that all sets share relatively many similarities in terms of the items included in communities showing the consistency of communities found by the modularity optimization method.

After this comparison of Set A and Set B, each of these smaller subsets has also been compared with the full data set to analyse the consistency amongst these sets further. Set A and the Whole data set also share a large number of similarities between the modularity communities found in each set as shown in Table 3. Community one is comprised of 14 of the same items, community two of the whole set and community three of Set A had 4 similarities, community three of the whole set compared with community two of Set A had 5, community four in each had 9, community five had 8, community six had 8, and community seven also had 8 of the same items in both data sets.

**Table 3:** Comparison of communities in Set A and Whole Data Set.

Community	*c*1W [14]	*c*2W [6]	*c*3W [15]	*c*4W [9]	*c*5W [8]	*c*6W [8]	*c*7W [9]
***c*** **1A [15]**	**14**	1	0	0	0	0	0
***c*** **3A [13]**	0	**4**	8	0	0	0	1
***c*** **2A [5]**	0	0	**5**	0	0	0	0
***c*** **4A [9]**	0	0	0	**9**	0	0	0
***c*** **5A [9]**	0	1	0	0	**8**	0	0
***c*** **6A [8]**	0	0	0	0	0	**8**	0
***c*** **7A [10]**	0	0	2	0	0	0	**8**

We can observe here that two communities (C4A and C4w) are identical, as it is also the case of C1w and C1A which are almost identical. The same happens with C5A and C5w. The numbers in parentheses indicate the number of items existent in each community or construct.

Similarities exist between Set A and Set B, Set A and the Whole Set, however, the amount of similarities between Set B and the Whole Data Set is lower but still has a similar dispersal of items amongst communities as shown in Table 4. The comparison of these two sets indicated that there are common items between communities; however these are lower than the comparison with Set A. Comparing community one between Set B and the whole set shows 6 common items, community two of the whole set and community three of Set B shows 5 common items, community three of the whole set and community two of Set B share 8 common items, community four in each have 5 similarities in terms of items, community five has four, community six has 6 and community 7 has seven of the same items. Furthermore, community eight of Set B has 5 items which are split between communities one and three of the whole set and Set B's community nine contains 4 items which are all part of community 5 of the whole set, as is the case in Set A. This perhaps indicates that some of the larger communities found in Set A and the Whole Set, have been further sub-divided in Set B whilst the individual items are still grouped together in a similar fashion.

**Table 4:** Comparison of communities in Set B and Whole Data Set.

Community	*c*1W [14]	*c*2W [6]	*c*3W [15]	*c*4W [9]	*c*5W [8]	*c*6W [8]	*c*7W [9]
***c*** **1B [6]**	**6**	0	0	0	0	0	0
***c*** **3B [10]**	2	**5**	2	1	0	0	0
***c*** **2B [15]**	3	0	**8**	3	0	1	0
***c*** **4B [5]**	0	0	0	**5**	0	0	0
***c*** **5B [6]**	0	1	0	0	**4**	1	0
***c*** **6B [6]**	0	0	0	0	0	**6**	0
***c*** **7B [12]**	0	0	3	0	0	0	**9**
***c*** **8B [5]**	3	0	2	0	0	0	0
***c*** **9B [4]**	0	0	0	0	4	0	0

The numbers in parentheses indicate the number of items existent in each community or construct.

**Figure 5:**
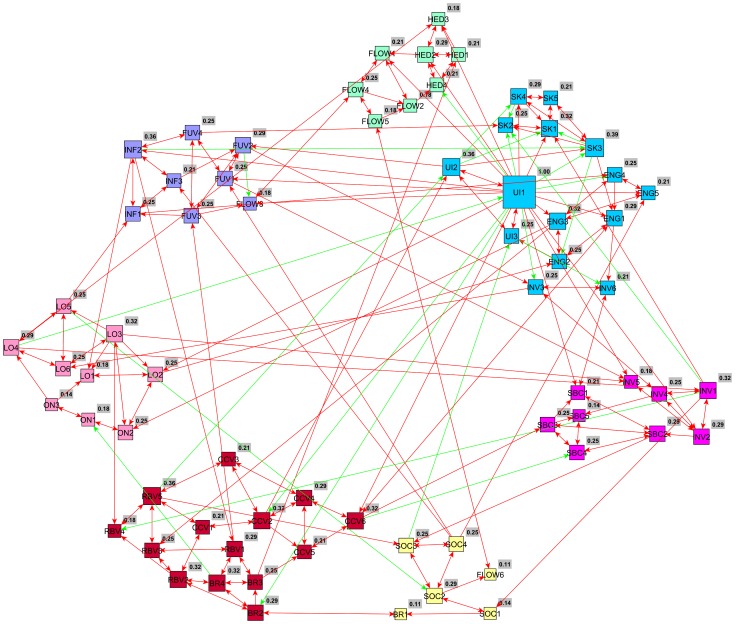
Whole Data Set – A network generated in the same manner as presented in Figure 3 and Figure 4. Here the majority of the Flow variables group together with all four Hedonic variables, as in Figure 3. Furthermore, most of the Brand Involvement and Self-Brand Congruency are grouped together, consistently with Figures 3 and 4. Again, the size of a node is proportional to its centrality in the network measured by ‘*node betweenness centrality*’ A green line represents a negative relationship found and a red line a positive relationship between the two nodes (items).

After conducting visual examination of the contingency tables, it becomes apparent that Set A and the Whole Data Set share more commonalities in terms of items as shown in the main diagonal (cells in boldface). This being said, the contingency tables in this section and the visualized graphs in Figures 3, 4 and 5 show there are still a large number of commonalities between all three data sets.

For a more thorough comparison of the communities found within each set, statistical analysis of level of agreement has been undertaken. As stated in our proposed methods, the *Adjusted Rand Index* (ARI) [50], *Fleiss' Kappa* [51] and *Cramer's V* [43] have been computed in order to compare the separation of the items into groups across the three different data sets. The ARI is a measure that ranges between zero and one, with zero indicating no agreement and one indicating perfect agreement. The ARIs for comparing the Data Sets are shown in Table 5. Set A and the Whole Set indicate the highest ARI value of 0.715, Set A and Set B have an ARI measure of 0.375 and Set B and the Whole Set 0.367.

**Table 5:** Adjusted Rand Index, Fleiss' Kappa and Cramer's V Results.

Comparison of Data Set	ARI	Kappa	Cramer's V
All Three Data Sets	n.a.	0.569	n.a.
Set A and Whole Set	0.715	0.76	0.863
Set B and Whole Set	0.367	0.381	0.762
Set A and Set B	0.375	0.566	0.766

Fleiss' Kappa was computed to compare Set A with Set B, Set A with the Whole Data Set, Set B with the Whole Data Set and all three compared at the same time. According to Landis and Koch's division [51], kappa values can be interpreted as follows: if the values are below zero they are consider to be of ‘*poor agreement*’, values between zero and 0.2 are regarded as in ‘*slight agreement*’, next, values larger than 0.2 and smaller than 0.4 is a ‘*fair agreement*’, next, values larger than 0.4 and smaller than 0.6 is a ‘*moderate agreement*’, next, values larger than 0.6 and smaller than 0.8 is a ‘*substantial agreement*’, and finally, values larger than 0.8 and smaller or equal to 1.0 is an ‘*almost perfect agreement*’.

The overall Fleiss' Kappa for comparing all communities in all three data sets was 0.569 as shown in Table 5, indicating a ‘moderate agreement’ of communities between the data sets. The Kappa computed for Set A and the Whole Set was the highest at 0.76 which means a substantial agreement is found between these two sets. The Kappa comparing Set A and Set B is lower at 0.566, however it still indicates a moderate agreement. Finally, the lowest Kappa computed was that comparing Set B to the Whole Data Set: 0.381 which indicates a fair agreement. Furthermore, the relatively high values of the Cramer's V tests indicate strong relationships between the corresponding communities found in each set. As displayed in Table 5 the Cramer's V for comparing Set A with the Whole Set equals to 0.863, comparing Set B with the Whole Set gives a Cramer's V value of 0.762 and finally the Cramer's V comparing Set A and Set B is 0.766. These statistical measures confirm our visual analysis of the contingency tables as they find strong relationships and associations across the communities found in each data set.

### Post-Hoc Analysis – Comparison of data-driven constructs versus theory-driven constructs

In order to further assess the constructs found by the novel methodology we propose here, each of the data-driven functional constructs (communities) is compared to the theory-driven constructs. Results of this comparison are displayed in Tables 6, 7 and 8 showing communities of items (*c*1A – *c*7A, *c*1B – *c*9B and *c*1W- *c*7W) for Set A, Set B and the Whole Set respectively. The numbers in parentheses indicate the number of items existent in each community or construct. The diagonal areas (showed in boldface) in each table show the majority of the similarities between communities of items and the literature-driven constructs. As can be seen in Tables 6, 7 and 8, the majority of the items derived from the literature-driven constructs have remained together although they have been combined with other items into a community of items found by the modularity optimization approach. However, some items that were part of the same literature constructs have been split up and assigned to different item communities. Some examples of this are those items relating to Flow, Co-Creation Value, Relationship-Building Value and, Brand Interaction Value and Brand Involvement which have been split in some instances and paired with items relating to other theoretical-driven constructs. These brand-related and brand-relationship oriented items were often paired together or were closely linked via arcs as shown in Figures 3,4 and 5 and shown by the split of these constructs in the Tables 6, 7 and 8. The theoretical meaning of the findings in these Tables is further discussed in the next section.

**Table 6:** Comparison of Modularity Communities in Set A with Literature-Driven Constructs.

	CCV [6]	ENG [5]	SK [5]	LO [6]	FLOW [6]	FUV [4]	SBC [5]	BR [4]	HED [4]	ON [3]	RBV [5]	SOC [4]	INF [3]	INV [6]	UI [3]
C1A [15]	**6**	0	0	0	0	0	0	4	0	0	5	0	0	0	0
C2A [5]	0	**5**	0	0	0	0	0	0	0	0	0	0	0	0	0
C3A [13]	0	0	**5**	0	0	0	0	0	0	0	0	4	0	1	3
C4A [9]	0	0	0	**6**	0	0	0	0	0	3	0	0	0	0	0
C5A [9]	0	0	0	0	**5**	0	0	0	4	0	0	0	0	0	0
C6A [8]	0	0	0	0	1	**4**	0	0	0	0	0	0	3	0	0
C7A [10]	0	0	0	0	0	0	**5**	0	0	0	0	0	0	5	0

**Table 7:** Comparison of Modularity Communities in Set B with Literature-Driven Constructs.

	CCV [6]	ENG [5]	SK [5]	LO [6]	FLOW [6]	FUV [4]	SBC [5]	BR [4]	HED [4]	ON [3]	RBV [5]	SOC [4]	INF [3]	INV [6]	UI [3]
C1B [6]	**0**	0	0	0	0	0	0	3	0	0	3	0	0	0	0
C2B [15]	1	**5**	3	0	1	0	0	0	0	3	2	0	0	0	0
C3B [10]	2	0	**0**	1	0	0	0	1	0	0	0	4	0	0	2
C4B [5]	0	0	0	**5**	0	0	0	0	0	0	0	0	0	0	0
C5B [6]	0	0	0	0	**5**	0	0	0	0	0	0	0	1	0	0
C6B [6]	0	0	0	0	0	**4**	0	0	0	0	0	0	2	0	0
C7B [12]	0	0	0	0	0	0	**5**	0	0	0	0	0	0	6	1
C8B [5]	3	0	2	0	0	0	0	**0**	0	0	0	0	0	0	0
C9B [4]	0	0	0	0	0	0	0	0	**4**	0	0	0	0	0	0

**Table 8:** Comparison of Modularity Communities in Whole Data Set with Literature-Driven Constructs.

	CCV [6]	ENG [5]	SK [5]	LO [6]	FLOW [6]	FUV [4]	SBC [5]	BR [4]	HED [4]	ON [3]	RBV [5]	SOC [4]	INF [3]	INV [6]	UI [3]
C1w [14]	**6**	0	0	0	0	0	0	3	0	0	5	0	0	0	0
C2w [6]	0	**0**	0	0	1	0	0	1	0	0	0	4	0	0	0
C3w [15]	0	5	**5**	0	0	0	0	0	0	0	0	0	0	2	3
C4w [9]	0	0	0	**6**	0	0	0	0	0	3	0	0	0	0	0
C5w [8]	0	0	0	0	**4**	0	0	0	4	0	0	0	0	0	0
C6w [8]	0	0	0	0	1	**4**	0	0	0	0	0	0	3	0	0
C7w [9]	0	0	0	0	0	0	**5**	0	0	0	0	0	0	4	0

Again, in order to support our visual inspection of these comparisons, a statistical measure; *Cramer's V* has been computed for each of the contingency Tables. Results of this statistical test are shown in Table 9. Comparing the communities, or ‘functional constructs’ found in Set A with the theoretically provided constructs gives a Cramer's V value of 0.971. For Set B compared to the theory driven construct a Cramer's V value of 0.860 is found and when computed for the Whole Set, Cramer's V equals 0.929. These relatively high values for Cramer's V indicate that this study based on a data-driven approach find mathematically supported ‘functional constructs’ that still have a high level of association with the constructs provided by theory. This may suggest that these findings are robust and provide a clear separation of the total 69 items of the questionnaire, rather than randomly being assigned based on correlation as is possible with any mathematical model based on correlations.

**Table 9:** Cramer's V Results for Functional vs. Theoretical Construct Comparison.

Comparison of Data Set	Cramer's V
Set A with Literature-Driven Constructs	0.971
Set B with Literature-Driven Constructs	0.860
Whole Data Set with Literature-Driven Constructs	0.929

### Stage 5 – Investigation of Directed Cycles and Common Feedback Loops

As a result of our investigation of models that can predict one variable from the other variables in the set we have produced a directed graph. As stated, it can be expected that the graphs generated in this study are not acyclic so it is natural to investigate the cycle structure since it may indicate any possible feedback loops. The baseline approach is to directly compare the common cycles present in the three data sets. However, we have soon discovered that this may lead to a computationally rather difficult task. As outlined in our proposed methodology, the graph size; the number of nodes and arcs, was reduced in safe manner in order to allow investigation of cycles.

However, even after the safe reduction of the number of nodes, and hence the number of arcs by the merging procedure, we have found an extremely large number of cycles in each graph through computational methods. For instance, in the ‘reduced’ graph for Set A, we computed a total of 6,227 cycles, for Set B, which has a larger number of vertices and accordingly adds to the combinatorial explosion, the number dramatically increased to 569,057 cycles; in the Whole Data Set we have 16,961 cycles. As the purpose of this paper was to propose a novel methodology of finding and modeling functional constructs, we choose to discuss the computational complexity issues that this may have for future research in the Discussion section in which we will make several recommendations according to our gained experience. In spite of this fact, in this contribution we do provide a comparison of the common arcs between the graphs of a reduced size in order to identify several small-scale cycles of interest and provide a basic understanding of, and outline the computational ‘hardness’ of the problem at hand regarding the detection of cycles. Table 10 depicts the comparison of the arcs between all three ‘reduced-in-size’ data sets and again shows that Set A and the Whole Set are overall more similar than Set B.

**Table 10:** Comparison of Arcs between Data Sets with safe reduction of the number of Arcs.

Comparison of Arcs	Number of Communalities
Common Arcs in Set A, Set B and Whole Data Set	20
Common Arcs in Set A and Set B	24
Common Arcs in Set A and Whole Set	39
Common Arcs in Set B and Whole Set	29
Common Arcs in Set A and Set B but not in Whole Set	4
Common Arcs in Set A and Whole Set but not in Set B	19
Common Arcs in Set B and Whole Set but not in Set A	9

Comparison of Arcs	Number of Dissimilarities
Arcs in Set A and not in Set B	41
Arcs in Set B and not in Set A	65
Arcs in Set A and not in Whole Set	26
Arcs in Set B and not in Whole Set	60
Arcs in Whole Set and not in Set A	32
Arcs in Whole Set and not in Set B	42

What is striking in this analysis is that, although the number of cycles is of the orders of thousands, the number of arcs that are common tend to be very small. We will also address this issue in the Discussion section where we will introduce another graph optimization problem, called ‘*topological network alignment*’ [52]. This combinatorial problem relates to this issue and we consider that it may have a key role in marketing and mathematical modeling of consumer behavior when iterative processes are inferred from data-driven research.

**Figure 6:**
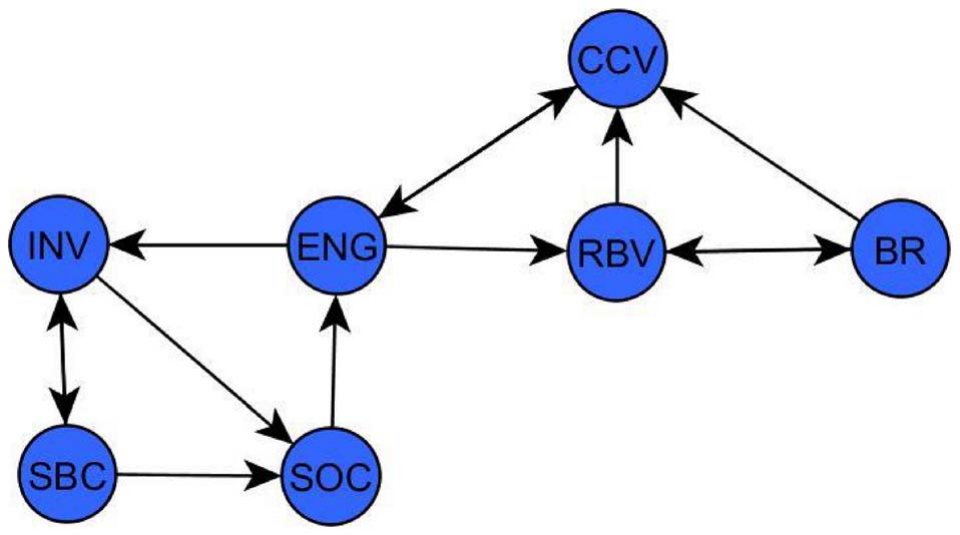
Positive cycles found in the ‘reduced’ Whole Data Set graph.

As stated, we provide some preliminary light on the issue of finding cycles in directed graphs relating to consumer behavior. Looking at the graph formed by reducing the amount of nodes and arcs in the Whole Data Set graph, we were able to easily inspect any possible cycles visually that encompassed the Customer Engagement item which, as mentioned before, has been the main variable of interest and the initial motivation in this study. The result of this visual inspection is shown in Figure 6 which depicts two different cycles that include the customer engagement item.

As shown in Figure 6, there are multiple small cycles and sub-cycles that encompass the customer engagement item. All of the relationships between these variables were of a positive nature indicating a positive ‘feedback loop’ to customer engagement. In this small graph there are two paths leading out of customer engagement; one indicating that Customer Engagement (ENG) has an effect on Brand Involvement (INV) and one indicating an effect from Customer Engagement on Relationship-Building Value (RBV). Furthermore, there are two paths leading back to customer engagement, one coming directly from Co-Creation Value (CCV) and one from Social Value (SOC) indicating a direct effect from these constructs on customer engagement.

## Discussion

### Implications for Theory

In this contribution, within the context of customer engagement in the online world, we have proposed a method to identify groups of questions (items), that have functional relationships which segregate them from others in a particular way. While it is always possible to produce a clustering of questionnaire responses using some particular correlation measure between the answers given by a set of respondents, here we propose that a model-driven approach may unveil functional relationships between the variables which can complement other pair-wise correlation studies of associations. Thus, this methodology aims to identify novel types of constructs from data. We have conducted our study using a questionnaire of customer engagement behaviors which was built up using sets of questions that belong to particular theoretically-driven constructs as a controlled setting for methodology development. As a consequence of the previous design of the questionnaire, it is expected that the profile of respondents for a pair of questions that belong to the same construct are naturally similar and naturally correlated. However, as we have observed, in some cases a particular profile of question responses can be more properly predicted by a construct involving up to three individual questions that do not belong to the same theory-driven construct. Motivated by data-driven research we aim at developing a novel methodology as displayed in Figure 1. In doing so, sets of constructs which are highly connected in our model-based directed network can be computed as the result of a process of community detection.

We have used an algorithm for community detection based on modularity optimization which is part of a free and online software tool for network visualization and manipulation. The results show that the emerging communities have a good level of agreement supported by our evaluation using statistical measures. The origin of the interrelation between variables is the result of either strong correlations between them (for example, as we may expect for variables of the same theoretical construct) or their joint predictive ability as input variables acting together in a model. The group predictive action on other sets of variables may suggest that these communities could be named as ‘*functional constructs*’ (which we propose as perhaps a short denomination from the lengthier: “data-derived functional constructs”).

Ideally, we would expect that the discovery of functional constructs across different data sets, conserved in different scenarios and studies, could lead to the development of better theoretical models for behavior analysis. Possibly this will require an iterative process in which reverse engineering from data can be incorporated as a necessary step within a consumer behavior model building, both informing each other, leading to iterative refinement of future models and data collection strategies. Collectively, the remaining “functional constructs” of this proposed endeavour; those that are invariant or very frequent across several data sets and problem domains, can be further analysed for expansion or refinement with new cohorts and particular survey designs.

Another specific implication to theory of our study is the grouping of a majority of Functional Value items and Informational Value items which jointly occur in all three cases. We have also observed that all the Customer Engagement items appear always together but in Set B and Whole Set they are combined with others as well. Perhaps this indicates that customer engagement could be measured more accurately by a combination of the items relating to the theoretically-driven construct of customer engagement as well as other measurement items. More specifically, in the graph produced by the whole data set, as shown in Figure 5, customer engagement items are combined with those items relating to a customer's usage intensity of the brand Facebook page and the customer's subjective knowledge of using the online social media platform. The subjective knowledge items were also linked to customer engagement in the graph resulting from Set B as shown in Figure 4.

Furthermore, the majority of items of the self-brand congruency and brand involvement constructs always appear together as shown in Figures 3, 4 and 5. This may indicate that a larger and more complex construct could be inferred from the data in order to more accurately measure the customers' previous relationship with a brand. For example, in their research on online consumer behaviors towards sports brands, Carlson and O'Cass [13] propose the construct ‘Brand Strength’ which in their study includes the concept of ‘Brand Involvement’ and ‘Brand Attitude’. Due to the experiments in this study consistently finding that the variables of brand involvement and self-brand congruency appear in one community, we propose that a possible larger ‘functional construct’, similar to the ‘Brand Strength’ construct brought forward by Carlson and O'Cass’ measures the consumer's perceived ‘strength’ or relationship with the brand more accurately.

Also, the items relation to functional value and informational value of the brand page consistently are grouped together in communities forming another possible ‘functional construct’ as indicated by the data and method in this study. Through mathematically finding this result consistently across all three data sets, we suggest that these constructs are not separate distinct constructs, but rather, form part of a larger ‘functional construct’ which measures a customer's perceived value of a branded social media page in terms of functionality and informational value combined.

Continuing this analysis, we see that the items relating to ‘flow’ never appear alone as a sole construct. In both in Set A and the Whole Set the majority of items relating to flow are found in the same ‘community’ (or functional construct) as all items of hedonic value. This finding provides an initial insight into a users' state of flow on a social media platform and the possibility of an important role of a customer's perceived hedonic value of the branded social media page in this process. Therefore, we argue that future research could further investigate the ties between and measurement of hedonic value of a brand's social media page and the possibility for a customer to achieve a ‘state of flow’ whilst using that page.

As discussed, the investigation of cycles or feedback loops within a graph provides an interesting but computationally hard problem. The example we presented in Figure 6 already provided theory with a few implications. For instance, we observe ENG leading to RBV, leading to BR, which leads to CCV and then back to ENG. As well as ENG leading to INV, leading to either SBC or SOC and then SOC back to ENG. It is thus very tempting to associate some temporal causal inference in terms of what is, after all, possibly the result of an iterative process by which consumers engage more when their perceived Co-Creation Value, relationship-building value or brand interaction value of the brand and the brand's social media page are higher. However, to provide conclusive results, future research in the area needs to investigate this issue further.

### Limitations

Possible limitations of this study are also identified as it is important to ensure clarity for our readers and future researchers. Firstly, after the random split of the Whole Data Set, we obtained Set A and Set B which were similar in terms of size and average age, however, examining the descriptive statistics of these two smaller sets further, there was a percentage difference of males and females within the sets which may have impacted upon the differences found later between two sets. Secondly, we recognize that the items included in the questionnaire of this study are not conclusive of all possible consumer behaviors to a brand which may inhibit the ability to ‘learn from data’ in this data-driven study. However, as shown by our initial discussion of extant literature on the topic, a number of varying views and studies were incorporated in generating and informing the survey which is why we argue this data set sufficient for the purposes of this study.

We also outline a few limitations regarding the methodology in this study. First, we have limited the computation time in the *Eureqa* software to be running for a few minutes, which is really not significant as we have observed that generally the variables in the best linear regression model do not change as a result of longer runs, but only the coefficients change slightly. This does not affect the uncovering of inter item relationships, only when the researcher is interested in the actual coefficient computed by *Eureqa* would this restriction become an issue. However, for the purpose of this study, we argue that this self-imposed ‘rule’ did not prove to restrict our findings.

Secondly; in this study we are only considering linear regression models to build the directed network and the whole process requires only a few minutes of computation using the *Eureqa* package. These combined limitations, however, are not harsh but we should list them and consider them in this discussion. The models selected are indeed the best that have been observed on the Pareto frontier of best models (as evolved by *Eureqa*) and, as a consequence, they are highly competitive in terms of model fitting when compared against the other non-linear models in the frontier.

### Future Research

As we provide a novel methodology in this study, possible areas for future research have been uncovered. Considering the data in this study was limited to those variables which were included as items in the questionnaire, future research in the area should consider including more consumer engagement related items, consumer behavior related items, or even investigate other aspects of interest such as consumers’ personalities and their effect on online customer engagement behaviors. Furthermore, future research could also include data obtained from actual social media platforms such as the actual number of shares, likes, and comments a customer makes on the branded social media page like the research of Cvijikj and Michahelles [4] included, in order to gain a greater understanding of that person's level of actual online engagement behaviors with the brand. Such research with a wide range of possible items available would be able to yield more conclusive results about actual online customer behaviors.

Secondly, we have identified several limitations of how we run the symbolic regression in this study and how the results were interpreted. An alternative, for future research, would be to use aggregated information (such as that described in Figure 2) with weights related to the probability of a variable to be in a model. This may help give an extra degree of flexibility as the current approach of deciding whether an arc is incorporated to the network would be less strict, thus marginally less good results from alternative models would not get completely ignored. An individual question item may be connected to a set of the most used questions in the models of the Pareto optimality frontier, regardless if the models are linear or non-linear. This may lead to a different methodology in which directed arcs, and now weights, should be considered as the final result and be exploited for reverse engineering purposes. This is left as an interesting avenue for future research.

Another future research topic comes to address the issue of comparing the results from each of the data sets; Set A, Set B and the Whole Set. As discussed, the large number of arcs and the extremely large number of cycles found in each set, make for a computationally hard problem. We suggest future research to address this problem using mathematical models such as those provided by the ‘*topological network alignment*’ and ‘*sub-graph isomorphism*’ problems. To justify our recommendation for future research, we briefly explain this problem and how exactly it can facilitate future research. In the well-known *sub-graph isomorphism problem*, a classical problem of discrete applied mathematics, we are giving an unweighted and undirected graph *G(V,E)* and we are asked if *G* would be an *exact* sub-graph of another graph *H(U,F)*. The *network alignment problem* thus generalizes the subgraph isomorphism problem. It it we are now required to find an optimal way to identify *a closely resembling* graph *G'* into *H* when the input graph *G* does not exist as an exact sub-graph of *H*. Behavioral scientists now armed with powerful algorithms for the network alignment problem would then have methods that can address the problem of identifying conserved sub-networks which allows dealing with natural variations in the networks generated from different cohorts. These algorithms may circumvent the need of reducing the graph by our heuristic approach of merging nodes of the same construct. Importantly, they will lead to fully-automatable solutions for inferring functional constructs directly from the data with less semantic and problem-domain knowledge.

Furthermore, in this initial study, we resorted to the free software tool *Gephi* and its way to compute communities using modularity as a quality function, but other objectives have been proposed and should be investigated. Several problems related to identification of community structure thus relying on heuristic algorithms for those cases in which it is unfeasible to find the optimal solution in a reasonable amount of computer time. Another suggestion for future research in order to identify functional constructs with other community detection algorithms would be to use a different modularity optimization methodology. As an example we can refer to the work of Gomez, Jensen and Arenas [53] who proposed a new formulation of modularity which allows the analysis of signed networks in general. Adopting such a method would allow for more advanced analysis in the future.

In sum, the proposed methodology is a step in a dialogue between computer science and human behaviour studies, based on discrete applied mathematics, with the emphasis in learning from data of consumer behavior and attitudes. We propose a five-stage novel methodology for finding data-driven ‘functional constructs’ in survey data. This study was conducted in the context of online customer engagement, however, as discussed, we envision the methodology of this paper to be adopted by researchers in various fields investigation human behaviours. Powered by the recent advances in heuristic and metaheuristic algorithms (i.e. memetic algorithms) [54], there is sure hope that they will become accepted and can handle large data sets. The identification of “functional constructs” would lead to a process of new mathematical modeling and perhaps as stated; motivate new study designs of human behavior.

## Supporting Information

File S1 **Survey Questionnaire Tool.**
(PDF)

File S2 **Responses Data.**
(XLSX)
