# Supplementary material for: A Data-Driven Approach to Reverse Engineering Customer Engagement Models: Towards Functional Constructs
Source: PLoS One. 2014 Jul 18;9(7):e102768. doi: 10.1371/journal.pone.0102768 (PMC4103885; doi:10.1371/journal.pone.0102768)
Supplement: File S1 — Survey Questionnaire Tool. (PDF) [file pone.0102768.s001.pdf]

Examining the antecedents and consequences of customer engagement in Facebook brand pages

THE UNIVERSITY OF NEWCASTLE AUSTRALIA

A Research Project conducted from the University of Newcastle

- The purpose of the following survey is to assess your attitudes and usage behaviours towards YOUR **favourite** Facebook Brand Page.
- It will take approximately **10 minutes** of your time to complete.
- You may withdraw from completing this questionnaire at any time without reason or penalty.
- Your name is not required and your responses are anonymous and will remain strictly confidential - **THIS SURVEY IS ANONYMOUS.**
- Your data will be securely stored at the University of Newcastle.
- **Instructions:**
  - ⇒ Please choose and write down your favourite brand's Facebook page.
  - ⇒ Then, answer all questions in full, even if some may appear similar.
  - ⇒ You are encouraged to take your time and complete the survey at your convenience.

All responses will be stored securely, and once the information has been analysed, all data will be securely stored and retained at the University of Newcastle for a minimum of five (5) years in accordance with the University policy.

Participation in this study is **entirely voluntary**. If you want to take part in the study, you are requested to complete the questionnaire in the attached booklet. When completed please hand the booklet back to the researcher.

Thank you for participating in the survey.
If you have any queries or require further information about the research, please contact:

Dr Jamie Carlson
University of Newcastle
Email: jamie.carlson@newcastle.edu.au

Or

Natalie de Vries
University of Newcastle
Email: c3099900@uon.edu.au

This research has been approved by the university's Human Research Ethics Committee (HREC). Approval Reference Number: H-2013-0226.
However, if you have any concerns about the manner in which this research is conducted please do not hesitate to contact the University's Human Research Ethics Officer, Research Branch, Chancellery, University of Newcastle, Callaghan NSW 2308.
Telephone (02) 4921 6333.

Section A: Picking your Favourite Facebook Brand Page

Q1. Please state your favourite Facebook brand page that you have regularly used in the past six months. (Please select one brand only)

....

Section B: Using your Favourite Facebook Brand Page

Please answer the following questions referring to the brand identified in Question 1:

Q2. Which of the following best describes how often you use the Facebook brand page:

☐ Daily ☐ Once a week or more ☐ Once a month or more

Q3. Approximately how long have you been a user of the brand's Facebook page? (please tick one)

☐ Less than a month ☐ 1 to 2 months ☐ 3 to 5 months ☐ 6 months or more

Q4. Approximately how often does the brand upload new content on its Facebook page? (please tick one)

☐ Less than once a week ☐ 1-3 times a week ☐ 4-6 times a week ☐ Daily ☐ 2-3 times a day ☐ 4 times a day or more

Q5. From what device do you MOSTLY access the brand's Facebook page? (please tick one)

☐ Home desktop PC ☐ Work PC (including a home office) ☐ Laptop ☐ Mobile telephone ☐ Mobile tablet
☐ Other: …………………..

Q6. Please rate the following statements on a scale of 1 (Strongly Disagree) – 7 (Strongly Agree) relating to your usage of the brand's Facebook page you nominated in Q1:

		Strongly disagree	Moderately disagree	Slightly disagree	Neither Agree nor disagree	Slightly agree	Moderately agree	Strongly agree
UI1	I frequently use the Facebook brand page	1	2	3	4	5	6	7
UI2	I often use the Facebook brand page	1	2	3	4	5	6	7
UI3	I regularly use the Facebook brand page	1	2	3	4	5	6	7

Q7. The following statements relate to YOUR level of skill and knowledge in using Facebook in general. On a scale of 1 (Strongly Disagree) – 7 (Strongly Agree), please rate the following questions:

		Strongly disagree	Moderately disagree	Slightly disagree	Neither agree nor disagree	Slightly agree	Moderately agree	Strongly agree
SK1	I know a lot about using Facebook	1	2	3	4	5	6	7
SK2	I feel knowledgeable about using Facebook	1	2	3	4	5	6	7
SK3	Among my circle of friends, I'm one of the "experts" on using Facebook	1	2	3	4	5	6	7
SK4	Compared to most other people, I know more about using Facebook	1	2	3	4	5	6	7
SK5	When it comes to using Facebook, I really know a lot	1	2	3	4	5	6	7

Section C: Relationship with the Brand

B. The following statements relate to YOUR favourite brand selected in Section A. NOT the Facebook page of that Brand. On a scale of 1 (Strongly Disagree) – 7 (Strongly Agree), please rate the following questions:

		Strongly disagree	Moderately disagree	Slightly disagree	Neither Agree nor disagree	Slightly agree	Moderately agree	Strongly agree
INV1	This brand means a lot to me	1	2	3	4	5	6	7
INV2	This brand is significant to me	1	2	3	4	5	6	7
INV3	I consider this brand to be a relevant part of my life	1	2	3	4	5	6	7
INV4	For me personally, this brand is important	1	2	3	4	5	6	7
INV5	I am interested in this brand	1	2	3	4	5	6	7
INV6	I am involved in/with this brand	1	2	3	4	5	6	7
SBC1	This brand is a lot like me	1	2	3	4	5	6	7
SBC2	This brand reflects what I am	1	2	3	4	5	6	7
SBC3	This brand is exactly how I see myself	1	2	3	4	5	6	7
SBC4	This brand image corresponds to my self-image in many respects	1	2	3	4	5	6	7
SBC5	Through this brand, I can express what I find important in life	1	2	3	4	5	6	7

Section D: Usefulness of the Brand's Facebook Page

C. The following statements relate to your overall assessment of the utility of the Facebook brand page (i.e. how useful and functional the page is). Please indicate to what extent you agree or disagree with the statements. On a scale of 1 (Strongly Disagree) – 7 (Strongly Agree), please rate the following questions:

		Strongly disagree	Moderately disagree	Slightly disagree	Neither Agree nor disagree	Slightly agree	Moderately agree	Strongly agree
FUV1	The content of the Facebook brand page is helpful for me	1	2	3	4	5	6	7
FUV2	The content of the Facebook brand page is useful for me	1	2	3	4	5	6	7
FUV3	The content of the Facebook brand page is functional for me	1	2	3	4	5	6	7
FUV4	The content of the Facebook brand page is practical	1	2	3	4	5	6	7
INF1	Information on the Facebook brand page is effective	1	2	3	4	5	6	7
INF2	The Facebook brand page adequately meets my information needs to make informed decisions	1	2	3	4	5	6	7
INF3	The Facebook brand page site provides in-depth information	1	2	3	4	5	6	7

Section E: Enjoyment of the Brand’s Facebook Page

D. i. The following statements relate to your overall assessment in the enjoyment YOUR favourite Facebook brand page provides to you. Please indicate to what extent you agree or disagree with the statements. On a scale of 1 (Strongly Disagree) – 7 (Strongly Agree), please rate the following questions:

		Strongly disagree	Moderately disagree	Slightly disagree	Neither Agree nor disagree	Slightly agree	Moderately agree	Strongly agree
HED1	The content of the Facebook brand page is fun	1	2	3	4	5	6	7
HED2	The content of the Facebook brand page is exciting	1	2	3	4	5	6	7
HED3	The content of the Facebook brand page is pleasant	1	2	3	4	5	6	7
HED4	The content of the Facebook brand page is entertaining	1	2	3	4	5	6	7

D. ii. There is a psychological state that can occur with people while they are undertaking certain tasks called ‘Flow’. This feeling occurs in many diverse activities, including sports, hobbies, work, Internet use and watching TV. In reference to the statement in bold below, please choose one answer only for each question:

When I am visiting (using/operating) my favourite Facebook brand page:

		Strongly disagree	Moderatel y disagree	Slightly disagree	Neither agree nor disagree	Slightly agree	Moderate ly agree	Strongly agree
FLOW1	I become totally involved in what I am doing	1	2	3	4	5	6	7
FLOW2	I lose track of time	1	2	3	4	5	6	7
FLOW3	I experience a feeling of "being in total control"	1	2	3	4	5	6	7
FLOW4	I feel immersed in what I am doing	1	2	3	4	5	6	7
FLOW5	I can escape from the everyday world	1	2	3	4	5	6	7
FLOW6	I experience a loss of self-consciousness	1	2	3	4	5	6	7

Section F: Social-Interaction of the Facebook Brand Page

E. The following statements relate to your overall assessment in the level of social-interaction YOUR favourite Facebook brand page provides to you. Please indicate to what extent you agree or disagree with the statements. On a scale of 1 (Strongly Disagree) – 7 (Strongly Agree), please rate the following questions:

		Strongly disagree	Moderatel y disagree	Slightly disagree	Neither Agree nor disagree	Slightly agree	Moderatel y agree	Strongly agree
SOC1	I can meet people like me on this Facebook brand page	1	2	3	4	5	6	7
SOC2	I can meet new people like me on Facebook brand page	1	2	3	4	5	6	7
SOC3	I can find out about people like me on this Facebook brand page	1	2	3	4	5	6	7
SOC4	I can interact with people like me on this Facebook brand page	1	2	3	4	5	6	7

Section G: Brand-Interaction of the Facebook Brand Page

F. The following statements relate to your overall assessment in the level of brand-interaction YOUR favourite brand's Facebook page provides to you. Please indicate to what extent you agree or disagree with the statements. On a scale of 1 (Strongly Disagree) – 7 (Strongly Agree), please rate the following questions:

		Strongly disagree	Moderately disagree	Slightly disagree	Neither Agree nor disagree	Slightly agree	Moderately agree	Strongly agree
BR1	I can interact with the brand on this Facebook brand page	1	2	3	4	5	6	7
BR2	I can communicate with the brand on this Facebook brand page	1	2	3	4	5	6	7
BR3	I can give feedback to the brand on this Facebook brand page	1	2	3	4	5	6	7
BR4	I can get answers from the brand on this Facebook brand page	1	2	3	4	5	6	7
RBV1	The Facebook brand page is committed to ensuring that I have easy access to the brand at any time	1	2	3	4	5	6	7
RBV2	The Facebook brand page is committed to ensuring rapid response standards to effectively deal with any enquiry I may have	1	2	3	4	5	6	7
RBV3	The Facebook brand page is committed to having continuing relationships with me	1	2	3	4	5	6	7
RBV4	The Facebook brand page is committed to delivering add-in values (e.g. special offers, member programs) to keep me loyal to the brand	1	2	3	4	5	6	7
RBV5	The Facebook brand page is committed to maintaining long term relationships with me	1	2	3	4	5	6	7
CCV1	The Facebook brand page interacts with me to serve me better	1	2	3	4	5	6	7
CCV2	The Facebook brand page works together with me to produce offerings that better suit me	1	2	3	4	5	6	7
CCV3	The Facebook brand page interacts with me to design offerings that meet my needs	1	2	3	4	5	6	7
CCV4	The Facebook brand page provides services in conjunction with me	1	2	3	4	5	6	7
CCV5	The Facebook brand page allows my involvement in providing services to me to get the experience that I want	1	2	3	4	5	6	7
CCV6	The Facebook brand page provides me with services that I help create	1	2	3	4	5	6	7

Section H: Your Engagement with the Facebook Brand Page

G. The following statements relate to you perception of your engagement with YOUR favourite Facebook Brand Page selected in Section A. Please indicate to what extent you agree or disagree with the statements. On a scale of 1 (Strongly Disagree) – 7 (Strongly Agree), please rate the following questions:

		Strongly disagree	Moderately disagree	Slightly disagree	Neither agree nor disagree	Slightly agree	Moderately agree	Strongly agree
ENG1	I am an integrated member of this Facebook brand page community	1	2	3	4	5	6	7
ENG2	I am an engaged member of this Facebook brand page community	1	2	3	4	5	6	7
ENG3	I am an active member of this Facebook brand page community	1	2	3	4	5	6	7
ENG4	I am a participating member of this Facebook brand page community	1	2	3	4	5	6	7
ENG5	I am an interacting member of this Facebook brand page community	1	2	3	4	5	6	7

Section I: Loyalty to the Brand

H. The following statements relate to your overall loyalty intentions with YOUR selected brand. Please indicate to what extent you agree or disagree with the statements. On a scale of 1 (Strongly Disagree) – 7 (Strongly Agree), please rate the following questions:

		Strongly disagree	Moderately disagree	Slightly disagree	Neither agree nor disagree	Slightly agree	Moderately agree	Strongly agree
LO1	I recommend this brand to other people	1	2	3	4	5	6	7
ON3	I will comment on this brand's Facebook content in the future	1	2	3	4	5	6	7
ON1	I will share this brand's Facebook page content in the future	1	2	3	4	5	6	7
LO4	I intend to remain loyal to this brand in the future	1	2	3	4	5	6	7
LO5	I will not stop supporting this brand	1	2	3	4	5	6	7
LO6	I think of myself as a loyal customer/supporter of this brand	1	2	3	4	5	6	7
LO3	I say positive things about this brand to other people	1	2	3	4	5	6	7
ON2	I intend to 'like' this brand's Facebook page content	1	2	3	4	5	6	7
LO2	I introduce this brand to other people	1	2	3	4	5	6	7

Section J: Demographic Information

I. Please choose one answer only for each question below.

Q1. Age: ………. years old

Q2. Gender: ☐ Male ☐ Female

Q3. Nationality: ……………………………

Q4. How long have you had a Facebook account? (Please tick one)

☐ 3 or more years ☐ 2 – 3 years ☐ 1 – 2 years ☐ 6 months – 1 year ☐ Less than 6 months

Q5. On average, how often do you access and use Facebook during the course of a day?
(Please tick one)

☐ I am signed in all the time ☐ More than 10 times ☐ 6 – 10 times ☐ 1 – 5 times ☐ Once daily ☐Rarely

Thank you very much for your time and care in completing this survey
